# Exclusive breastfeeding practices reported by mothers and the introduction of additional liquids

**DOI:** 10.1590/0104-1169.0141.2553

**Published:** 2015

**Authors:** Alessandra Marcuz de Souza Campos, Camila de Oliveira Chaoul, Elenice Valentim Carmona, Rosângela Higa, Ianê Nogueira do Vale

**Affiliations:** 1 Undergraduate student in Nursing, Faculdade de Enfermagem, Universidade Estadual de Campinas, Campinas, SP, Brazil. Schorlarship holder from Programa Institucional de Bolsas de Iniciação Científica (PIBIC), Brazil; 2 RN graduated in Faculdade de Enfermagem, Universidade Estadual de Campinas, Campinas, SP, Brazil. Schorlarship holder from Programa Institucional de Bolsas de Iniciação Científica (PIBIC), Brazil; 3 Post-doctoral fellow, School of Nursing, University of Texas, Health Science Center, San Antonio, TX, United States. Professor, Faculdade de Enfermagem, Universidade Estadual de Campinas, Campinas, SP, Brazil. Schorlarship holder from Fundação de Amparo à Pesquisa do Estado de São Paulo (FAPESP), Brazil; 4 PhD, RN, Hospital da Mulher "Prof. Dr. José Aristodemo Pinotti", Centro de Atenção Integral à Saúde da Mulher, Universidade Estadual de Campinas, Campinas, SP, Brazil; 5 PhD, Professor, Faculdade de Enfermagem, Universidade Estadual de Campinas, Campinas, SP, Brazil

**Keywords:** Breast Feeding, Drinking, Knowledge, Mothers, Infant Nutrition, Weaning

## Abstract

**Aim:**

To assess the concept of exclusive breastfeeding held by nursing women by comparing the period they consider that they perform it and the infants' age at the introduction of additional liquids.

**METHOD:**

Cross-sectional descriptive study conducted with 309 women who delivered babies at a university hospital in the interior of São Paulo, Brazil. The data were subjected to descriptive analysis; the variables of interest were crossed using the non-parametric Kruskal-Wallis test, the chi-square test and Fisher's exact test.

**RESULTS:**

Approximately 30% of the women reported having introduced additional liquids before the infants reached aged six months old, while asserting that they were performing exclusive breastfeeding. The following variables were associated with early introduction of liquids: lack of employment (p = 0.0386), younger maternal age (p = 0.0159) and first pregnancy (p = 0.003).

**CONCLUSION:**

The concept of exclusive breastfeeding might not be fully clear to women, as they seem to believe that it means not to feed the children other types of milk but that giving other liquids is allowed. These results show that promotion of breastfeeding should take beliefs and values into consideration to achieve effective dialogue and understanding with mothers.

## Introduction

Exclusive breastfeeding (EB) means that an infant receives only breast milk from his or her mother or a wet nurse, or expressed breast milk, and no other liquids or solids, with the exception of oral rehydration solution, drops or syrups consisting of vitamins, minerals supplements or medicines^(^ 1 ^)^.

The World Health Organization (WHO) recommends EB until the age of six months, and then to start adequate and safe complementary feeding of other liquids and foods, with continued breastfeeding up to 2 years of age or beyond^(^ 1 ^)^. One study conducted with 34,366 infants in Brazilian state capitals and the Federal District found that the prevalence of EB increased in recent decades from a median duration of 23.4 days in 1999 to 54.1 days in 2008^(^ 2 ^)^. However, regarding the prevalence of six-month EB as recommended by the WHO, much improvement is still needed. 

Feeding additional liquids (water, tea, fruit juice, etc.) to breastfed infants under six months old is a common practice; however, even when sporadically performed, this practice might result in reduced breast milk intake and consequent decreases in milk extraction and production, which might contribute to effects such as premature weaning, lesser weight gain and increased risk of diarrhea^(^ 1 ^,^ 3 ^-^ 4 ^)^. Several diseases are associated with non-EB, such as "necrotizing enterocolitis, diabetes, allergies, pneumonia, among others"^(^ 4 ^)^. Mothers might believe that feeding liquids other than milk before the age of six months is innocuous and helps to solve problems such as colic, infant gas, or even thirst^(^ 3 ^)^.

One study conducted at Basic Health Units (BHUs) in Rio de Janeiro (n= 1,507 breastfeeding mothers) found that maternal characteristics such as age, previous experience with breastfeeding and marital status are related with feeding liquids other than milk to infants under six months of age^(^ 3 ^)^.

According to the authors' clinical experience, some mothers believe they are performing EB even when they report giving their babies additional liquids and eventually also solid food concomitantly. This fact suggests that nursing women do not properly understand the concept of EB, which might be related with their difficulty to maintain EB until six months of age. 

The aim of the present study was to assess the understanding of the concept of EB by nursing women by comparing the period they consider that they perform EB and the infant's age at the introduction of additional liquids and food. 

## Method

The data for the present cross-sectional descriptive study were collected from June 2010 to June 2011. All of the women admitted to a rooming-in unit and who had previously delivered a child at a teaching hospital in the interior of the state of São Paulo were included in the study, while the mothers who had delivered twins or whose infants exhibited problems requiring intensive care for any duration were excluded. As concerns ethical issues, the text of the informed consent form was read, and the study aims were explained to the participants, including full assurance of anonymity, along with their right not to participate in the study without this affecting the care they would receive at the institution. The study was approved by the research ethics committee of the State University of Campinas (Universidade Estadual de Campinas - Unicamp), ruling no. 773/2008 and CAAE 0616.0.146.000-08.

A database was elaborated using Excel(r) that included the mothers' sociodemographic data (age, marital status, educational level, occupation, number of pregnancies and deliveries) the infants' birth data (birth weight, gestational age, gender and route of delivery), breastfeeding-related variables (durations of total and exclusive breastfeeding) and infant's age at introduction of additional liquids and food (water, tea, other types of milk, fruit juice, and others). 

Based on the comparison of variables "duration of EB" and "age at introduction of liquids as reported by the interviewed women", three categories were created: (1) true EB (no simultaneous use of any other liquid); (2) EB plus the use of other types of milk in addition to the breast milk; and (3) EB and concomitant feeding of liquids of any type. Thirteen forms were inconsistent due to missing data and were excluded from analysis; therefore, the final sample consisted of 296 forms.

The duration of EB was established in the present study based on the answers the mothers gave to the following questions posed at the beginning of the interviews: "How long did you breastfeed your child after discharge from the rooming-in unit"?, "Throughout the period of breastfeeding, did you also feed your child other liquids/food in addition to breast milk?", and "How old was your child when you first introduced additional liquids/food?" The question on the duration of EB was posed again at the end of the interview. 

Descriptive analysis was performed next. The quantitative variables corresponding to the three aforementioned categories were compared by means of the non-parametric Kruskal-Wallis test^(^ 5 ^)^ to investigate the presence of significant associations among them. In the cases in which the null hypothesis on the Kruskal-Wallis test was rejected, the posttest^(^ 6 ^)^ formulated by author Patrick Giraudoux was performed using the kruskalmc procedure in the pgirmness package of R 2.15.0. 

The chi-square test^(^ 5 ^)^ was used to investigate possible associations between the aforementioned categories and sample-related variables (mothers' socioeconomic and infants' birth data); that test was used to investigate associations between two categorical variables. Whenever at least 20% of the cells had expected counts of less than 5, Fisher's exact test was used^(^ 7 ^)^. The significance level was set to 5% in all of the analyses, which were performed using the statistical software SAS (*Statistical Analysis Software*), version 9.2^(^ 8 ^)^.

## Results

Data were collected from 309 women, and 296 were included for statistical analysis. Despite all care to ensure the greatest possible precision of the information on EB duration and infant's age at the introduction of liquids other than breast milk collected at different moments along the interviews, some forms had to be excluded from analysis due to missing data that did not allow for classification under any of the categories used to compare EB duration and the introduction of liquids other than breast milk. 

Most of the interviewees had attended elementary school only, had a stable partner, had no employment, and had given birth to healthy newborn infants by vaginal/forceps delivery, as Table 1, below, shows relative to the initial sample consisting of 309 women. 

**Table 1 -:** Distribution of the interviewed women per educational level, marital status, occupation, route of delivery, and child's gestational age. Campinas, SP, Brazil, 2011

Characteristics		n	%
Educational level			
	Elementary school	182	60.5
	Secondary school	107	35.5
	Higher education	12	4.0
	Total	301	100
Marital status			
	Married	254	83.3
	Single	51	16.7
	Total	305	100
Occupation			
	Employed	103	34.6
	Unemployed	195	65.4
	Total	298	100
Route of delivery			
	Vaginal/forceps	212	68.6
	Cesarean section	97	31.4
	Total	309	100
Gestational age			
	Full/post-term	300	97.1
	Preterm	9	2.9
	Total	309	100

The average age of the women was 22.2 years old (ranging from 13 to 37 years old; median = 22 years old). On average, they had two pregnancies (median = 1), 1.7 deliveries (median = 1) and had attended 8.7 prenatal visits (median = 9). The average duration of EB was 4 (± 2.3) months, thus falling short of the reported time for the introduction of additional food, which was 4.4 (± 2.6) months on average. Table 2, below, describes the quantitative variables assessed in the initial sample consisting of 309 women.

**Table 2 -:** Distribution of the interviewed women per age, number of pregnancies, deliveries and prenatal visits, duration of exclusive breastfeeding, and infants' age at introduction of additional liquids. Campinas, SP, Brazil, 2011

Variable	n	Mean	SD*	Minimum	Q1	Median	Q3	Maximum
Age	309	22.2	4.8	13	19	22	25	37
No. pregnancies	309	2	1.3	1	1	1	3	8
No. deliveries	287	1.7	1.1	0	1	1	2	8
No. prenatal visits	246	8.7	3.3	0	7	9	11	18
Exclusive breastfeeding duration (months)	307	4	2.3	0	2	4	6	24
Age at administration of liquids^†^	302	4.4	2.6	0	3	4	6	24

* Standard deviation;

† in months

**Figure 1 -:**
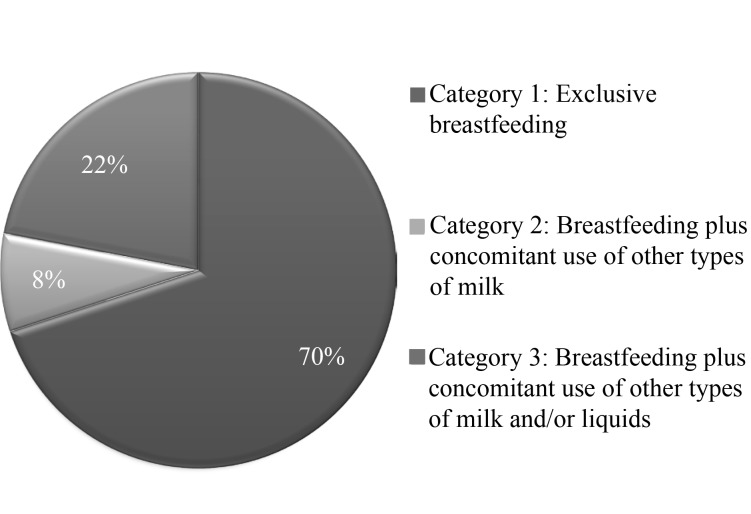
Categories resulting from the comparison of the duration of exclusive breastfeeding as reported by the mothers and the addition of other liquids to the children's diet. Campinas, SP, Brazil, 2011

Most interviewees (70%) reported to have fed the infants breast milk only during the EB period, while 30% reported to have introduced additional liquids, despite stating that they performed EB: 8% reported the introduction of other types of milk, and 22% reported the introduction of liquids of any type (categories 1, 2 and 3, respectively), as Figure 1, below, shows: 

The types of liquids the mothers reported giving their children during the alleged EB period were as follows: water (18.6%), other milks (17.9%), tea (13.8%), fruit juice (10.7%) and other liquids (3.5%). Comparison of the aforementioned categories to the qualitative variables revealed that "occupation" was associated with possible misunderstanding of the concept of EB (p = 0.0386). Introduction of other types of milk was most frequent among the women without employment (77.3%), although those women reported to have performed EB. Table 3, below, describes the comparison between categories and qualitative variables.

**Table 3 -:** Comparison of qualitative variables to the categories resulting from the comparison between the duration of exclusive breastfeeding as reported by the mothers and the addition of other liquids to the children's diet. Campinas, SP, Brazil, 2011

Variable		Category 1			Category 2			Category 3		p-value
		n	%		n	%		n	%	
Educational level										0.7481*
	Elementary	117	57.9		13	59.1		42	65.6	
	Secondary	77	38.1		8	36.4		19	29.7	
	Higher	8	4		1	4.6		3	4.7	
Marital status										0.5077^†^
	Married	168	81.6		20	90.9		54	84.4	
	Single	38	18.5		2	9.1		10	15.6	
Occupation										0.0386^†^
	Employed	63	31.8		5	22.7		31	47	
	Unemployed	135	68.2		17	77.3		35	53	
Route of delivery										0.6068^†^
	Vaginal/forceps	139	66.8		14	63.6		48	72.7	
	Cesarean section	69	33.2		8	36.4		18	27.3	
Gestational age										0.1451*
	Full-/post-term	203	97.6		20	90.9		65	98.5	
	Preterm	5	2.4		2	9.1		1	1.5	

* p-value on Fisher's exact test;

† p-value on the chi-square test

There was a significant association between maternal age and the addition of other liquids concomitantly to breastfeeding (p = 0.0159): younger mothers tended to introduce other liquids earlier. Similarly, the association between number of deliveries and the addition of other types of milk concomitantly to breast milk was also significant (p = 0.003): the introduction of other types of milk occurred earlier among the primiparous mothers, as Table 4, below, shows: 

**Table 4 -:** Comparison of quantitative variables to the categories resulting from the comparison between the duration of exclusive breastfeeding as reported by the mothers and the addition of other liquids to the children's diet. Campinas, SP, Brazil, 2011

Variable	Cat*	n	Mean	SD^†^	Min^‡^	Q1	Median	Q3	Max^§^	p-value^||^
Age	1	208	22.2	4.8	13	19	22	25	37	0.0159
	2	22	20	4.5	15	17	19	21	34	
	3	66	22.9	5	14	19	23	25	36	
No. pregnancies	1	208	2	1.4	1	1	1	3	8	0.0703
	2	22	1.4	0.7	1	1	1	2	3	
	3	66	1.8	1.1	1	1	1	2	5	
No. deliveries	1	197	1.8	1.2	1	1	1	2	8	0.0030
	2	21	1.1	0.4	0	1	1	1	2	
	3	58	1.7	1	1	1	1	2	5	
No. prenatal visits	1	165	8.8	3.2	0	7	9	10	18	0.0906
	2	21	7.3	3.6	0	5	8	10	13	
	3	51	9.2	3.2	0	7	10	12	14	

* Category;

† Standard deviation;

‡ Minimum;

§ Maximum;

|| p-value on the Kruskal-Wallis test

## Discussion

The number of pregnancies and of deliveries exhibited significant differences between their mean and median values: 2 and 1 (pregnancies) and 1.7 and 1 (deliveries), respectively. These findings show that there is a range of these variables, and consequently, larger numbers of both women pregnant for the first time and primiparous mothers, which denotes a greater influence of these women on the results relative to categories two and three. 

Even while claiming to perform EB, 30% of the interviewees reported feeding their children other liquids in addition to breast milk, which suggests a lack of understanding of the EB concept. 

These findings are supported by the results of other studies; for example, one study conducted in Horizonte, Ceará, with 120 parturient women found that although 89% of the participants admitted that the proper duration of EB is six months, 14% of them contradictorily stated that water and tea ought to be given also; 4.2% made additional mention of fruit juice^(^ 9 ^)^. Similarly, another study (performed with 50 mothers in the interior of São Paulo) found that most interviewees believed that giving liquids and fruit before age six months old is appropriate independently from breastfeeding^(^ 10 ^)^. One study conducted with 1,057 mothers from Laos, Southeast Asia, to assess the prevalence of and the factors that influence the use of breast milk (BM) substitutes provides further information on the limitations to EB; 20% of the participants reported having introduced formula and non-dairy products before six months of age ^(^ 11 ^)^.

In the present study, women without employment reported feeding their infants other types of milk before six months of age more often compared to formally employed women. This finding disagrees from the results of other studies^(^ 11 ^-^ 13 ^)^, which found that staying at home was a positive factor, i.e., favorable to successful breastfeeding. Thus, one might hypothesize that the women without employment are less informed and more susceptible to environmental influences. Those mothers, along with the primiparous mothers (possibly less experienced) are more prone to believe that feeding other (non-human) types of milk is necessary or that it does not interfere with EB. One study conducted with 48 puerperal women from Uberaba, Minas Gerais, found that 14.6% of the mothers believed that EB is impossible, for which reason complementary feeding using milks other than BM is justified^(^ 14 ^)^.

Although primiparous mothers are more prone to engage in breastfeeding, they usually tend to maintain the practice over a shorter time^(15) ^and to introduce complementary feeding earlier^(^ 13 ^)^.

The addition of various types of liquids along the period the interviewees mistakenly believed to be of EB exhibited a possible association with younger maternal age. This finding is corroborated by the results of other studies^(1,12-13) ^and suggests that as a function of their lack of experience, young mothers are more easily influenced by relatives as concerns practices detrimental for breastfeeding^(^ 13 ^)^.

Several factors might account for the decision of mothers to start complementary feeding. The results of qualitative studies conducted in Maranguape (Ceará, Brazil)^(^ 16 ^)^ and Cali (Cauca Valley, Colombia)^(^ 17 ^)^ and of a quantitative study (n=120) performed in Horizonte (Ceará, Brazil)^(^ 9 ^)^ include some of the mothers' beliefs invoked to justify the premature introduction of liquids and food, including "lack of milk", "weak milk" and that breast milk does not suffice to satiate the child's hunger or thirst. Those studies further found that the mothers were influenced by the children's grandmothers to introduce other types of food at the time when breastfeeding ought to be exclusive; that was particularly the case of the primiparous mothers, due to their lack of experience^(^ 9 ^,^ 16 ^-^ 17 ^)^.

The aforementioned qualitative study^(^ 17 ^)^ found that cultural beliefs might either favor (beliefs such as breast milk makes the children stronger and more intelligent and favors the mother-child affective bond) or hinder (beliefs and misgivings such as children remain hungry when they are given breast milk only; sunlight dries the breasts; children must be given other types of milk to avoid becoming "attached" to the mother's breasts; previous poor breastfeeding experiences involving nipple sores; infants should be given substances like "bean extract", which fortifies and/or cleanses their stomachs, among others) breastfeeding. Thus, breastfeeding is a cultural practice encompassing multiple meanings and behaviors in different communities^(^ 17 ^)^.

Behavior is not only influenced by intellectual knowledge but also by the beliefs and cultures present in the women's actual environment. For that reason, knowledge does not always guarantee that breastfeeding will be adequately performed. 

Although the sample of the present study included women that had previously delivered at least one child at a maternity hospital known for promoting breastfeeding, mistakes were detected in the actual practice of EB as reported by the interviewees. Such mistakes included premature and inappropriate introduction of other types of milk and/or liquids before six months of age. 

Mothers might possibly believe that giving liquids before six months of age is innocuous and solves problems such as colic, infant gas or even thirst^(^ 3 ^)^. A recent review published by The *Cochrane* Library on the introduction of additional food and liquids to full-term infants did not locate any study demonstrating that giving liquids is beneficial for newborn infants, in addition to describing possible risks^(^ 18 ^)^ that are discussed below.

In the present study, water was the liquid most often given by the mothers; the reason might be the mothers' belief that they should offer the children water to quench their thirst, although according to the WHO, several studies have shown that healthy infants do not need additional water during the first six months if they are exclusively breastfed, even in a hot climate^(^ 1 ^,^ 18 ^)^. Thus, one might infer that for mothers, milk is food suitable to satiate the infants' hunger (but not their thirst), while water is indispensable to keep them well hydrated. 

The risks associated with the addition of liquids and food to the diets of infants under six months old include the following: reduction of breast milk intake (with the consequent reductions of all associated benefits); lower milk production (resulting from reduced milk extraction); higher odds of shortening the duration of breastfeeding; difficulty establishing efficacious breastfeeding; and reduction of the mother's confidence with parallel reinforcement of the negative belief that BM is insufficient^(^ 1 ^,^ 18 ^-^ 19 ^)^. In addition, premature complementary feeding exposes infants to food or device contamination (i.e., bottles and nipples), in addition to the risk of reactions to non-human proteins or food dyes in processed foods. The latter risk is further reinforced by the reduced intake of antibodies contained in breast milk and the infant's immaturity vis-à-vis the digestion of complex substances^(^ 1 ^,^ 16 ^,^ 18 ^)^.

Premature complementary feeding is associated with an increased occurrence of anemia, infectious diseases (gastrointestinal and respiratory ones in particular), and growth problems^(^ 13 ^)^. In addition, complementary feeding might also have effects on mothers, including breast engorgement, mastitis, faster return to fertility, impaired mother-child bond and financial effects^(^ 18 ^)^.

Stability of the parental bond has been described as a positive influence on EB^(^ 1 ^,^ 13 ^)^. Attendance of higher education and performance of more than six prenatal visits are factors associated with better knowledge on breastfeeding, which tends to favor its practice ^(^ 12 ^-^ 13 ^,^ 15 ^)^, while a low educational level is considered to be an unfavorable factor^(^ 13 ^)^. Nevertheless, the number of prenatal visits (p = 0.0906), marital status (p = 0.5077) and educational level (p = 0.7481) of the participants in the present study seemed to exert neither positive nor negative influences on EB. 

## Conclusion

The present study found that the practice of EB as reported by a part of the participants had some elements of confusion or was the fruit of a mistaken idea on what exclusive breastfeeding actually means. 

Younger, primiparous mothers and/or those without employment are possibly the ones with more difficulties engaging in EB, with water and non-breast milk being the liquids most frequently given. 

Although it is not possible to assert that women poorly understand the concept of EB, the findings call attention to the need for further studies on strategies to improve the understanding of EB. 

## Final considerations

The present study represents a contribution to clinical practice, as it might stimulate healthcare professionals to reflect on both the comprehension of EB and the fact that its practice does not only involve information but also different cultural beliefs. On those grounds, nurses must strive to develop skills to identify the beliefs associated with the practices of nursing women on an individual basis and which of those beliefs might or should be preserved, changed, restructured or given new meanings. 

Acceptance by professionals of women's intellectually or culturally based previous knowledge will doubtlessly favor adherence to their recommendations. In addition, professionals must recognize that EB demands an effort from mothers, as the difficulties are above the desire to introduce additional food before the infant reaches six months of age. Professionals also should be aware that crisis periods interfere with the women's self-confidence relative to breastfeeding; thus, they must be ready to help the mothers, especially during such occasions. 

With this awareness, professionals can invest in strategies to promote EB, using scientific foundations to educate adults and to establish receptive environments where women, their partners and relatives can share their beliefs, doubts and feelings. 

One limitation of the present study arising from its retrospective nature is that the data on the age at which infants began to be fed additional liquids and food depended exclusively on the mothers' recollection. Therefore, further (prospective) studies ought to be performed to assess the women's understanding of EB, necessarily including the cultural beliefs associated with its practice.

## References

[1] World Health Organization (WHO) (2009). Infant and young child feeding: model chapter for textbooks for medical
students and allied health professionals.

[2] Venâncio SI, Escuder MML, Saldiva SRM, Giugliani ERJ (2010). A prática do aleitamento materno nas capitais brasileiras e Distrito
Federal: situação atual e avanços. J Pediatr.

[3] Niquini RP, Bittencourt SA, Lacerda EMA, Oliveira MIC, Leal MC (2010). Acolhimento e características maternas associados à oferta de líquidos
a lactentes. Rev Saúde Pública.

[4] Parizoto GM, Parada CMGL, Venâncio SI, Carvalhaes MABL (2009). Tendência e determinantes do aleitamento materno exclusivo em crianças
menores de 6 meses. J Pediatr.

[5] Pagano M, Gauvreau K (2004). Princípios de Bioestatística.

[6] Siegel S, Castellan NJ (1988). Non parametric statistics for the behavioural sciences.

[7] Mehta CR, Patel NR (1983). A network algorithm for performing Fisher's exact test in rxc
contingency tables. JASA..

[8] (2008). SAS/STAT(r) User's Guide, Version 9.2.

[9] Nogueira CMR (2009). Conhecimento sobre aleitamento materno de parturientes e prática de
aleitamento cruzado na Unidade Hospitalar e Maternidade Venâncio Raimundo de Sousa
- Horizonte - Ceará [Internet].

[10] Valezin DF, Ballestero E, Aparecido JC, Ribeiro JF, Marinho PCM, Costa LFV (2009). Instrumento educativo sobre alimentação de lactentes - baseado nas
necessidades de conhecimento das mães. Rev Inst Ciênc Saúde.

[11] Barennes H, Empis G, Quang TD, Sengkhamyong K, Phasavath P, Harimanana A (2012). Breast-Milk Substitutes: A New Old-Threat for Breastfeeding Policy in
Developing Countries. A Case Study in a Traditionally High Breastfeeding
Country. PLoS One.

[12] Sanches MTC, Buccini GS, Gimeno SGA, Rosa TEC, Bonamigo AW (2011). Fatores associados à interrupção do aleitamento materno exclusivo de
lactentes nascidos com baixo peso assistidos na atenção básica. Cad Saúde Pública.

[13] Silva VMM, Joventino ES, Arcanjo DS, Veras JEGLF, Dodt RCM, Oriá MOB (2009). Conhecimento de puérperas acerca da amamentação - estudo
descritivo. Online Braz J Nurs.

[14] Fonseca MO, Parreira BDM, Machado DC, Machado ARM (2011). Aleitamento materno: conhecimento de mães admitidas no alojamento
conjunto de um hospital universitário. Cienc Cuid Saude.

[15] Volpato SE, Braun A, Pegorim RM, Ferreira DC, Beduschi CS, Souza KM (2009). Avaliação do conhecimento da mãe em relação ao aleitamento materno
durante o período pré-natal em gestantes atendidas no Ambulatório Materno Infantil
em Tubarão, (SC). Arq Catarin Med.

[16] Frota MA, Casimiro CF, Bastos PO, Sousa OA Filho, Martins MC, Gondim APS (2013). Mothers' knowledge concerning breastfeeding and complementation food:
an exploratory study. Online Braz J Nurs.

[17] Hernández L, Vásquez ML (2010). Practices and beliefs about exclusive breastfeeding by women living in
Commune 5 in Cali, Colombia. Colomb Med.

[18] Becker GE, Remmington S, Remmington T (2011). Early additional food and fluids for healthy breastfed
full-terminfants. Cochrane Database of Systematic Rev..

[19] Silva L, Elles M, Silva M, Santos I, Souza K, Carvalho S (2012). Social factors that influence breastfeeding in preterm infants: a
descriptive study. Online Braz J Nurs.

